# The Microstructure and Cracking Behaviors of Pure Molybdenum Fabricated by Selective Laser Melting

**DOI:** 10.3390/ma15186230

**Published:** 2022-09-08

**Authors:** Anru Yan, Abbas Mirza Atif, Xiaobo Wang, Tian Lan, Zhiyong Wang

**Affiliations:** 1 Institute of Laser Engineering, Beijing University of Technology, Beijing 100124, China; 2 Institute of Atomic and Molecular Science, Shaanxi University of Science & Technology, Xi’an 710021, China

**Keywords:** selective laser melting, pure molybdenum, densification, microstructure, cracks

## Abstract

Selective laser melting (SLM) of pure molybdenum encounters all the difficulties of SLM metals due to its intrinsic properties (high melting point, high ductile-to-brittle transition temperature and high surface tension). In this work, we studied the influence of key factors such as powder morphology and processing parameters on SLM fabricated pure molybdenum. Pure molybdenum with a relative density of 99.1% was fabricated by SLM using optimized processing parameters. The formation mechanisms for densification behavior and crack growth behaviors are systematically analyzed. Electron backscattered diffraction analysis indicates that the interlocking grain boundary structure and stretch columnar grains can increase bonding force and inhibit crack growth. The balling and cracking can be reduced by adding support structure and suppressing oxygen content. The hardness of SLM-fabricated molybdenum exceeding 260 HV, which is 30–37% higher than Mo prepared by conventional manufacturing methods, mainly attributed to the fine grains and dislocation strengthening in the SLM process. The bending strength of SLM-ed Mo reached 280 ± 52 Mpa. The fracture mode of SLM Mo was intergranular. This study provides a new route for the fabrication of refractory metals with a complex structure.

## 1. Introduction

Refractory materials like molybdenum, with exceptional intrinsic properties (high melting point, high tensile strength and low thermal expansion coefficient), are a suitable application for high-temperature, high-reliability components in harsh environments, such as electrical package devices [1] and plasma-facing components [2]. These parts usually have precision construction, i.e., molybdenum can be used as a heat sink material for chips, due to its low thermal expansion coefficient and high thermal conductivity. It can be used as a metal oxide semiconductor gate in very large integrated circuits, and mounting the IC on molybdenum eliminates the “bimetal effect”; it can also be used as protective sheets for converter armor in nuclear fusion reactors [3]. Molybdenum, and its alloys, are usually manufactured by powder metallurgy; there are limitations in the possibility of manufacturing parts with complex geometries, and in small batches.

Selective laser melting (SLM) technology is a manufacturing method perfectly suited to produce highly complex parts in small and individualized batches. SLM uses a high energy density laser as the energy source. It can produce three-dimensional metal components with complex outer and inner geometries from powders in one step, without the need for part-specific tooling or preproduction costs. The process of SLM is rapid melting and solidification, resulting in the formation of fine grains and a dense structure. The advantages of SLM fabricated parts are: high machining accuracy and resolution; high material availability; freedom of part design and production flexibility; and excellent mechanical properties [4]. Due to these irreplaceable advantages, SLM technology is increasingly being used in the manufacturing of precision parts such as tool blades, with conformal cooling channels and functional parts with high geometric complexity, ideal for applications in electronic equipment, medical equipment, aerospace components and lighting. However, to date, the production of fully dense and crack-free molybdenum parts with adequate mechanical properties via SLM, have seldom been reported. Electron Beam Melting (EBM) is another additive manufacturing technology, which has a similar process compared to SLM, except that EBM uses an electron beam of energy. At present, EBM has good application in the forming of superalloy and high melting point metals. However, since the beam spot diameter of EBM is usually one order of magnitude larger than that of SLM technology, the result of EBM parts is usually Ra 25~35 μm. The SLM result is Ra 3~12 μm, thus SLM has more advantages with regards to detail characteristics and the complexity of parts. Jonathan Wright et al. compared the formation of refractory metal tungsten using SLM and EBM technology. The results showed that thermal cracks were generated both in SLM and EBM fabricated tungsten samples: further increasing the preheating temperature and cabin temperature of SLM would help to suppress the development of cracks; and the high vacuum degree and high construction temperature of EBM could reduce the generation of cracks. However, the authors believe that SLM is preferred for applications where mechanical properties are critical, and complex geometry is required. Zhao et al. compared microstructure and the mechanical properties of SLM and EBM Ti–6Al–4V samples; the results showed that the tensile strength of SLM formed titanium alloy samples was significantly higher than that of EBM samples. The part size has a significant effect on the microstructure and mechanical properties of the EBM samples, but less on the SLM samples [5].

It has been shown that materials well suited to SLM, such as titanium alloy [6], nickel alloys [7], aluminum alloy [8], cobalt alloys [9] and copper alloy [10], have good laser absorption and a good balance of melting point, thermal conductivity, surface tension, instinctive brittleness and viscosity. Mechanical properties of such metals fabricated by SLM are better than those manufactured by traditional methods. However, the major challenge in selective laser melting of molybdenum is the formation of cracks and pores. According to research findings, the SLM of molybdenum encounters almost all intractable difficulties of metal SLM [11]; the high melting point (2893K) results in high cohesive energy [12] and promotes balling behavior, which results in open porosity [13,14]. In addition, the oxidation sensitivity may reduce wettability [15,16] and results in high brittleness and high viscosity [17], further lowering the fracture toughness, which also results in Mo susceptible to cracking in high temperature forming, ultimately restricting the feasibility and limiting the application [2,18]. Thus, fundamental investigations are necessary to assess the application of SLM technology to refractory materials [14,19]. Some literatures have studied the inhibition of crack development in refractory metals by adding alloying elements; cracks are not completely eliminated and the addition of alloying elements can cause other problems. When adding 0.5 wt% ZrC in another refractory metal-tungsten, grain size can be significantly reduced, resulting in improved crack resistance and an 88.7% reduction in crack density [15]; however, grain size reduction will weaken the thermal conductivity, which would limit the application of Mo in electronic packaging. The classification of defects by monitoring the powder laying state of SLMs has been investigated [20]. This study helps to identify some defects quickly, and if it can be further extended to detect the influence of temperature, stress and other influencing factors, on crack formation, it will help the rapid and accurate detection of defects.

The purpose of this study is to fabricate pure Mo parts by SLM and to investigate the key factors of densification and cracking formation. Densification, microstructure, cracking behavior and mechanical performance are studied in detail. The results show that cracking in refractory metals can be suppressed, or even eliminated, by controlling the temperature and adding support structure. Our work will provide support for the application of Mo in high-performance fields such as electronics and nuclear industry.

## 2. Materials and Methods

### 2.1. Material

**Figure 1:**
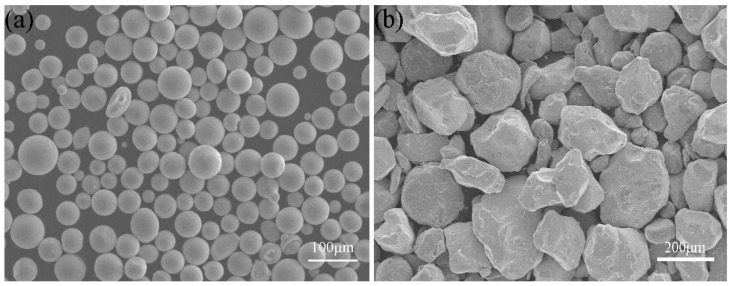
SEM micrograph of molybdenum powder: (**a**) spheroidized powder, (**b**) polygon powder.

The raw materials used in this study were plasma spheroidized molybdenum powder (Tekna Advanced Materials Inc, Sherbrooke, QC, Canada) and polygon molybdenum powder (Jinchun Inc, Chengdu, China), and the purity was 99.9%. The morphology of two kinds of molybdenum powder are shown in Figure 1. The trace elements of spheroidized molybdenum powder were provided by Tekna Advanced Materials Inc., as shown in Table 1.

### 2.2. Experimental Procedure

The SLM experiments were conducted on an EOS M100 machine equipped with an IPG 200 W ytterbium fiber laser, having a 40 μm focused laser beam diameter and the wavelength of 1064 nm (EOS Gmbh, Krailling, Germany). The blade of the recoating powder layer was a brush re-coater. The machine was also equipped with a preheating module that can preheat the baseplate up to 80 °C. All experiments were conducted in a flowing Argon atmosphere with a resulting oxygen content lower than 100 ppm in the working module.

**Figure 2:**
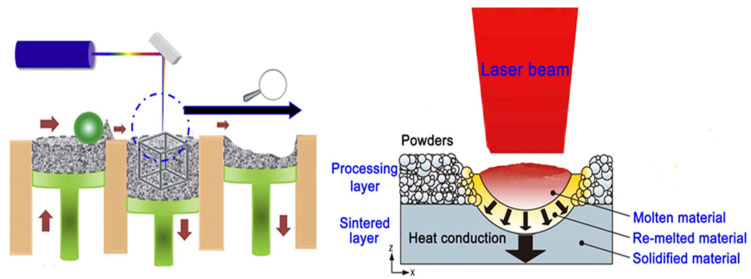
The processing principle of SLM technology.

The laser parameters were orthogonally designed, in which the laser power (P) and scanning speed (v) were 120–170 W and 50–550 mm/s, respectively. The laser scanned in a “meander” exposure type with 67° rotation between two adjacent layers; some studies have shown that 67° rotation facilitates the minimization of residual stresses. The overlap rate of the adjacent scanning line was 0–20%. The processing principle of SLM technology is shown in Figure 2.

### 2.3. Characterization

Powder fluidity was tested by Holzer flow meter (YTN-102, Judeantai, Tech.Co., Beijing, China). Samples of 8 × 8 × 8 mm^3^ were fabricated on a steel baseplate and removed by wire electrode cutting. The density of the samples were measured by the Archimedes method, according to ASTM B962-08 standard. Theoretical densities 10.2 g/cm^3^ for pure molybdenum were used to calculate the relative density. The surface morphology, microstructure and defect observations were performed using a Zeiss Merlin field emission scanning electron microscope (FE-SEM)(Zeiss, Oberkochen, German), equipped with an electron backscatter diffraction detector (EBSD) (Oxford Instruments, Oxford, UK) and HKL Channel 5 software (Oxford Instruments, Oxford, UK) packages. The molybdenum specimen for microstructural characterization were etched in standard Murakami′s solution (1:1:10 for KOH: K_3_[Fe (CN)_6_]: H_2_O). A microhardness test was carried out according to GB/T4340. 1-1999. The hardness value testing equipment was Shimadzu HMV-2T micro-Vickers Tester (SHIMADZU Instruments, Kyoto, Japan) and the test applied load was 50 gf for 10 s. The hardness value was an average value and the corresponding standard deviations were derived from five measurements. The ambient temperature 3–point bending test was carried out according to DIN EN ISO 3327. The load was applied in the building direction. A tensile test was completed on a universal testing machine, and fracture morphology was observed by FE-SEM (Zeiss, Oberkochen, German).

## 3. Results

### 3.1. Powder Characteristics for Forming Precision

As molybdenum has broaden application in high-end fields such as electronic package, aerospace and medical applications, which usually demand fine structures, the dimensional precision is thus particularly important as relative density. In previous studies on SLM forming W-Cu alloys, we found that the powder morphology has a direct effect on the forming capacity. Considering both economy and practicality, the influence of powder characteristics on forming precision and relative density were studied.

During the SLM process, both the spherical and polygon powders have a relatively uniform surface during the initial layers of SLM, while after several layers the polygon powder spread shows ripple-like fluctuations on the surface. As tested by a Holzer flow meter, the fluidity of spherical and polygon powders was 6 s/50 g and 13.5 s/50 g.

**Figure 3:**
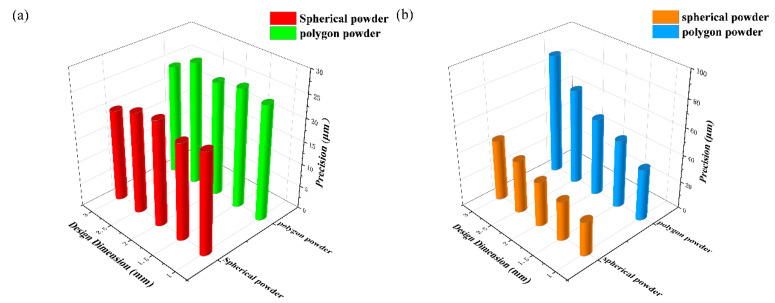
Absolute precision of Mo sample formed by SLM: (**a**) xy-plane direction, (**b**) z-direction.

A comparison of the dimensional precision of the samples, after forming two kinds of raw powder, is shown in Figure 3. The dimension in the xy-plane direction (scanning plane) was basically the same as the design value, and dimension precision is about 20–25 μm, while in the z-direction (building direction) there was a slight shrinkage, and the shrinkage of the polygon powder was two times that of the spherical powder.

A further comparison of the densities of the two types of powder-formed specimens showed that the density of the polygon powders (84.7%) was nearly 15% lower than the density of the spherical powders (99.1%). The phenomenon was similar to the work of Zhou et al. [21], which investigated the use of irregular powders to form pure tungsten and found that the density was only 86%.

**Figure 4:**
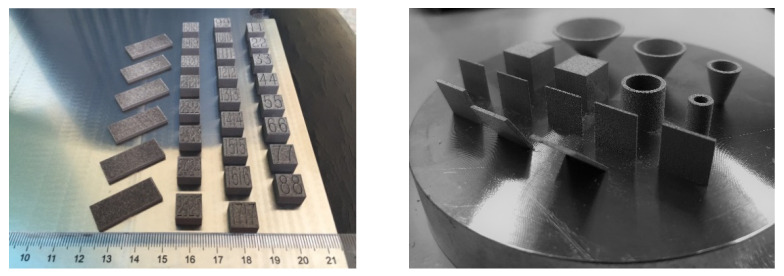
The SLM fabricated parts built up by orthogonal process parameter.

The surface morphology of specimens, and parts built up by the orthogonal process parameters, are shown in Figure 4. After the optimized parameters were analyzed, pure molybdenum specimens with different thicknesses, tilt angles and tapers, were further formed by the SLM technique, which can be seen in Figure 4, with excellent forming effect.

### 3.2. Process Parameters for SLM Molybdenum

**Figure 5:**
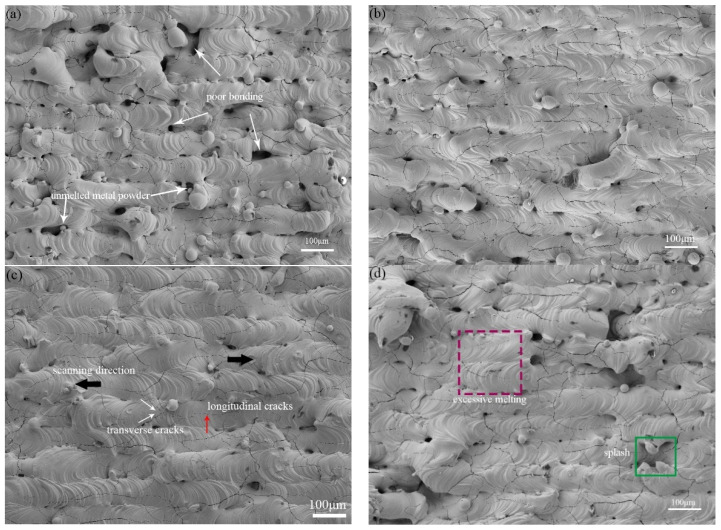

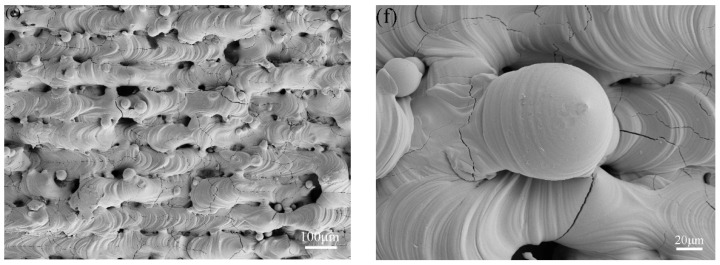
The melt track of the as-built Mo SLM parts under different zone: (**a**) zone A, LED = 0.36 J/mm, (**b**) zone B, LED = 0.64 J/mm, (**c**) zone C, LED = 0.51 J/mm, overlap = 20%, (**d**) zone D, LED = 0.80 J/mm, (**e**) LED = 0.51 J/mm, overlap = 0%, (**f**) balling phenomenon.

**Figure 6:**
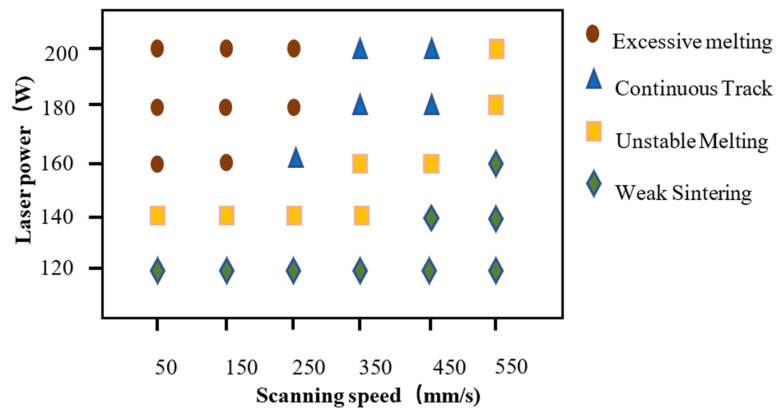
The process window of SLM Mo.

**Figure 7:**
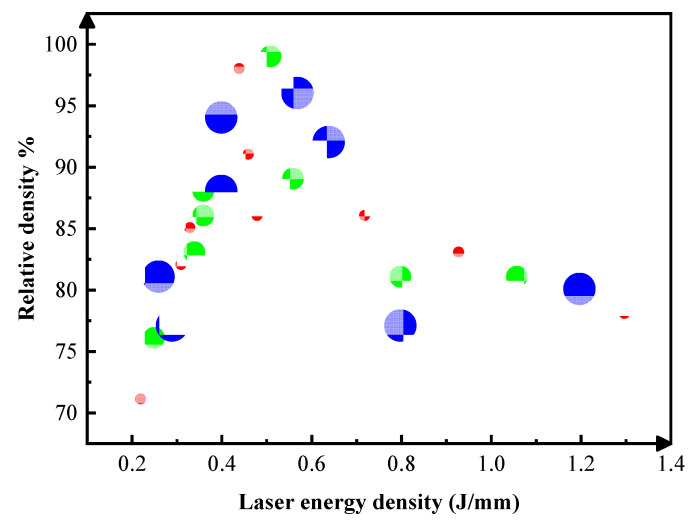
Relative density of SLM Mo as a function of the applied laser linear energy.

As illustrated in Figure 5, the SLM fabricated molybdenum bulk exhibits different morphology of molten tracks. The laser scanning direction (SD) can also be deduced according to the surface ripples (as shown by black arrows in Figure 5c). As shown in Figure 5, the processing window determined by the most important parameters (laser power and scanning velocity) could be divided into four different zones, depending on the quality of the melting tracks, including weak sintering zone (A), unstable melting zone (B), continuous track zone (C) and excessive zone (D). The process window, in Figure 5, revealed that the selected raw powder materials and processing optimization could overcome, in some degree, the difficulty of the high melting point and the viscosity of molybdenum. Different zones correspond to diverse linear energy density (LED), which was defined as LED = P/v (J/mm). In zone A, the scanning track was hard to form as the LED was insufficient; there is obviously irregular poor bonding defects, as shown in Figure 5a. In zone B, as shown in Figure 5b, the tracks were unstable due to insufficient energy; there is a lot of unmelted powder sticking to the surface. In zone C, the melt flow of the molten pool becomes stable, with sufficient depth to penetrate the front layer, resulting in a relatively smooth track being obtained, with an LED interval of 0.44 J/mm to 0.64 J/mm, as shown in Figure 6. When an excessive LED was applied, as seen in zone D, the surface pores were increasing obviously caused by the burning loss and splash. In Figure 6, the process window of molybdenum also showed that there were three main types of defects: (i) non-melted powder, (ii) voids, and (iii) cracks. At the over-fusion zone, the melt pool was as wide as twice the low energy one. Droplet spattering could be seen due to violent fluctuations and evaporation in the melt pool caused by Marangoni convection. Figure 7 shows the relative density of SLM Mo as a function of the LED. The maximum relative density of 99.1% was obtained at LED of 0.51 J/mm. It is worth noting that a different density may be obtained with the same LED. Accordingly, laser energy density is a parameter which is helpful for the rapid screening of processing parameters, but it cannot replace the analysis of the interaction between laser and materials.

The influence of the overlap rate was investigated at a constant LED = 0.5 J/mm (P = 200 W, v = 400 mm/s), as shown in Figure 5c,e. The surface morphology at 20% overlap rate (o = 20%) was uniform and the distribution of defects was almost regular, but the surface structure at 0% overlap rate showed massive irregularities and a significant increase in porosity. This was due to the energy of the laser spot in Gaussian distribution; the energy at the edge of the molten pool was weakened. At an overlap rate of 0%, lack of energy at the edge of the pool resulted in high surface tension, which meant the molten pool could not spread out and formed an arched track. This prevented the next layer from being deposited correctly, with poor bonding between layers. However, an apparent crack network can be observed on the surface, as indicated in Figure 5c. Cracks generally come in two directions: those parallel to the scanning direction are called longitudinal cracks (red arrow); and cracks perpendicular to the ellipse were defined as transverse cracks (white arrows). In the top view cross-section of SLM Mo, the longitudinal cracks grew along the center line of the molten pool, while the transverse cracks appeared in pairs, both vertical to the ellipse molten track, forming a “V” shape.

### 3.3. Microstructure

**Figure 8:**
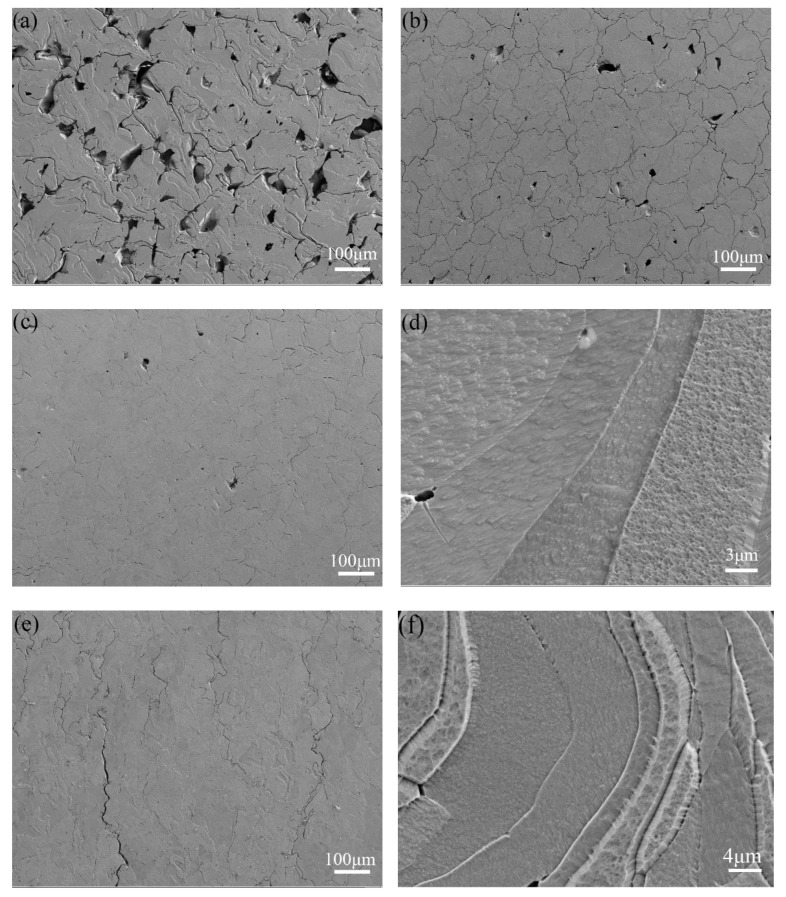
The SEM image of a-d top view and e-f side view cross section of SLM Mo: (**a**) top view of LED = 0.36 J/mm, (**b**) top view of LED = 0.64 J/mm, (**c**) top view of LED = 0.51 J/mm, (**d**) high-magnification of (**c**), (**e**) side view of LED = 0.51 J/mm, (**f**) high-magnification of (**e**).

Figure 8 shows the microstructural morphologies taken from horizontal and longitudinal cross-sections of SLM-fabricated Mo. As shown in Figure 8a–c, the cracks and micropores were suppressed with the increase of LED from 0.36 J/mm to 0.64 J/mm. The dense microstructure and some cracks were observed, as shown in Figure 8b. However, the pores and cracks increase gradually, with a further increase of LED. Grain boundaries could be clearly observed from the high-magnification SEM image, seen in Figure 8d, with massive nanosized spot-like sub-grains presented inside the grains. Some microcracks could be seen in the side view of Mo samples (Figure 8e). In Figure 8f, the high-magnification SEM image shows that elongated flame-like grains were observed, with some columnar crystals grown through several sintered layers.

### 3.4. Mechanical Performance

**Figure 9:**
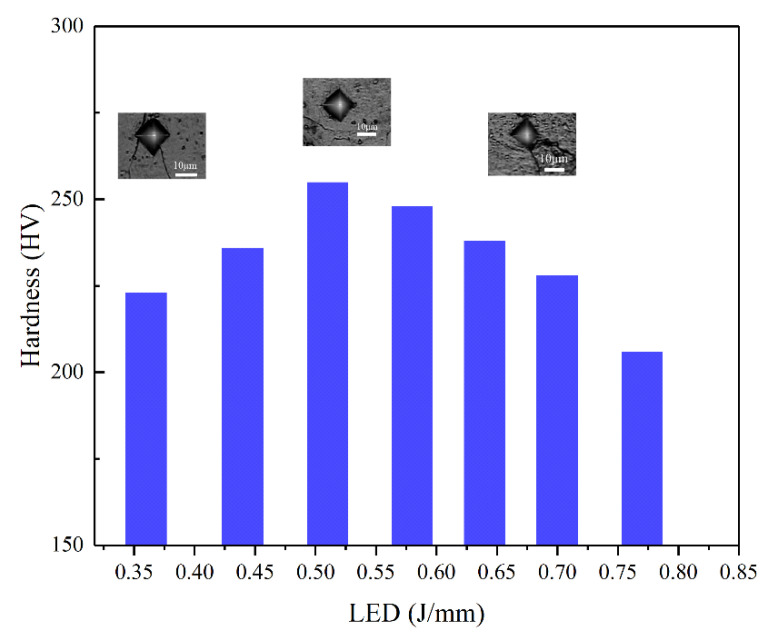
The effect of laser energy density on microhardness of SLM fabricated molybdenum.

Figure 9 illustrates the functional relationship between microhardness and LED; the hardness was measured from the cross-sections of SLM-ed pure Mo samples. The microhardness of the samples were in the range of 202 HV to 260 HV; the maximal hardness was obtained by the sample with LED of 0.51 J/mm.

**Figure 10:**
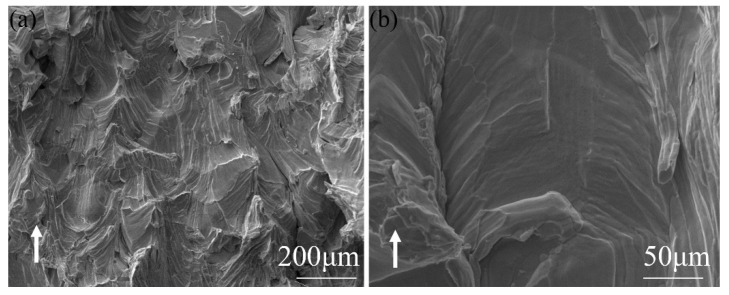
SEM image of fracture surface perpendicular to the building direction of Mo. (**a**) low-magnification, (**b**) high-magnification.

The bending strength of SLM-ed Mo reached 280 ± 52 Mpa. The fracture surface of molybdenum in different magnifications are given in Figure 10. The white arrow is the building direction of the sample. The fracture mode is almost exclusively intergranular.

## 4. Discussion

### 4.1. Densification Process

**Figure 11:**
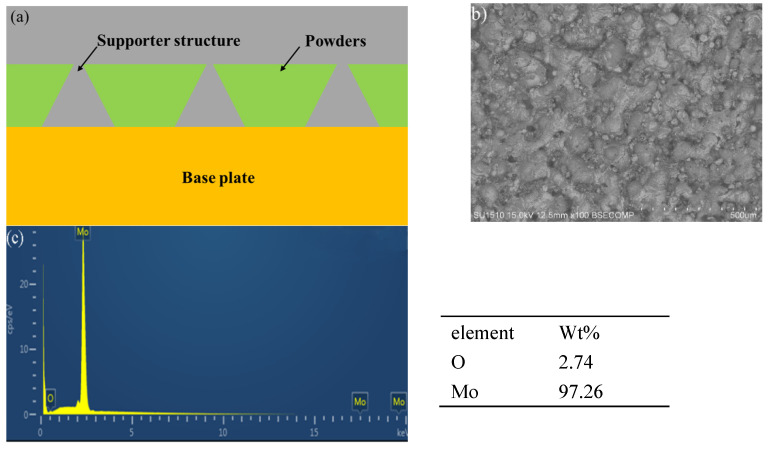
(**a**) the morphology of support structure, (**b**) top face morphology of Mo sample after adding support, (**c**) the EDS analysis of element content of SLM fabricated Mo samples.

The powder characteristics and process map are the key factors determining the densification mechanism. Spherical powder with high flowability and a smooth surface was beneficial to spread evenly and obtain homogeneous sintering. Meanwhile, the stack form of spherical powder could also increase laser absorptivity and improve wettability, in comparison to polygon powder [22,23]. As shown in Figure 11, the spherical powder layer was able to form multiple scattering, generating a large amount of additional laser absorption within the powder layer, and the incident beam was trapped in the powder particle gap, strengthening the interaction between the adjacent powder particles [24]. In addition, the Gaussian distribution of particle size improves the compaction density of the powder, which promote densification during the SLM process. The interval of Gaussian distribution also plays an important role in energy absorption and conversion. On the one hand, due to fluctuations in charge density, grain boundaries can facilitate the energy conversion of incident photons into the AC lattice [25]; this dictates that what SLM requires is a large number of small powders with as many grain boundaries as possible. On the other hand, due to the van der Waals force, smaller particles are more likely to clump together, reducing powder flowability and causing poor powder deposition. In our study, the molybdenum powder was well selected with a relatively reasonable powder size distribution, with D50 and D90 values of 23.8 μm and 34.4 μm. From the result of density and dimensional precision, this is a reasonable particle size distribution interval.

In process map, the main causes accounting for densification behavior in the four distinguishing zones are different. As shown in Figure 5, in weak sintering zone (A) and unstable melting zone (B), the key factor accounting for densification behavior is the liquid phase volume. The lower laser energy decreases the molten pool temperature, the result in the volume of liquid phase was insufficient to completely fill the voids between the un-melted Mo particles, and a large number of pores are formed in the Mo-Mo bonding area [26]. Therefore, when the density gradually increases with the LED at this stage, we can speculate that at the LED of 0.5 J/mm there is sufficient liquid phase maximizing the density. In the continuous track zone (C), the densification is significantly influenced by the dynamic of Marangoni flow in the molten pool. The intensity of the Marangoni flow increases with increasing LED, which also leads to the probability of gas dragging towards the melt pool and in turn leads to the formation of pores in the solidified pool [27]. Therefore, in the continuous track zone (C), as the LED increased from 0.5 J/mm to 0.7 J/mm, it causes increased porosity instead.

In addition to these aspects, the densification behavior can also be affected by the balling phenomenon. During the SLM process of Mo, the spread time of droplet is nearly twice the solidification time [21]. The velocity of the solidification front is obviously faster than that of droplet spreading. Therefore, the melt droplets could be arrested and solidify as a molten globule, which was observed in Figure 5f. When the laser scans such beads, the movement of the molten pool front undergoes a significant disturbance. Consequently, it is difficult to completely fill the inter-ball pores and results in density limitation. Meanwhile, the formation of incomplete melted powders in the molten pool could decrease the flowability and wettability, which also increases the porosity. Through adjusting the power and speed during laser selective melting, the melting and spreading time can be changed. In this work, keeping the laser parameters in the continuous track zone (C) was useful to give enough time for molybdenum melting and spreading; the balling phenomenon caused by rapid solidification was inhibited, allowing densities up to 10.10 g/cm to be achieved.

### 4.2. Crack Generation and Suppression

The unavoidabe cracking observed in Mo SLM-ed parts is the result of two main reasons. Firstly, cracks imply non-equilibrium solidification characteristics, the result of thermal stress arising during rapid solidification or recrystallization [28]. In the SLM process, the ultra-high cooling rate of laser processing could reach the order of 1010 K/s. The high thermal conductivity also contributes to the high cooling rate, thus, the newly deposited layer cools and shrinks on the established cool substrate; therefore, it can be concluded that cracks are initiated under tensile stress during the cooling process. Considering that the laser can provide so much heat during scanning, a supporting structure was designed to preserve the heat, as shown in Figure 11a. The supports should be fabricated at a low density by adjusting the laser parameters to the minimum heat loss rate. With the supports, the bottom of the sample (the grey part in Figure 11a) was surrounded by powders (the green part in Figure 11a), which has low thermal conductivity, thus, the thermal dispersion of the sample was controlled to a rather low level. The supports kept the sample at a high temperature, which was necessary to control cracks for SLM Mo. With this design, the SEM image in Figure 11b illustrates that the cracks were effectively suppressed, meanwhile, the surface morphology was smooth and balling phenomenon was alleviated. At the same time, the preheating function of the equipment also helps to suppress the cracks. The combination of the two auxiliary methods is an effective method to suppress the crack development.

Secondly, the intrinsic properties of molybdenum such as high brittleness, high oxidation sensitivity and low wettability, also promote the formation of such cracks. The ductile-to-brittle transition temperature (DBTT) is as high as 375 K. As the temperature drops below the DBTT, the residual stress rises above the yield strength in the brittle material, causing crack formation. Besides, the EDS analysis (Figure 11c) shows that the presence of impurities, such as a small amount of oxygen in SLMed Mo, also leads to the increase of DBTT, which decreases the surface energy and initiates fracture. Therefore, lower oxygen content in the process chamber and the original powder is essential to restrain crack. We will continue to study the effect of reducing oxygen content on cracks in our follow-up work.

### 4.3. Microstructure, Hardness and Fracture Mechanism

**Figure 12:**
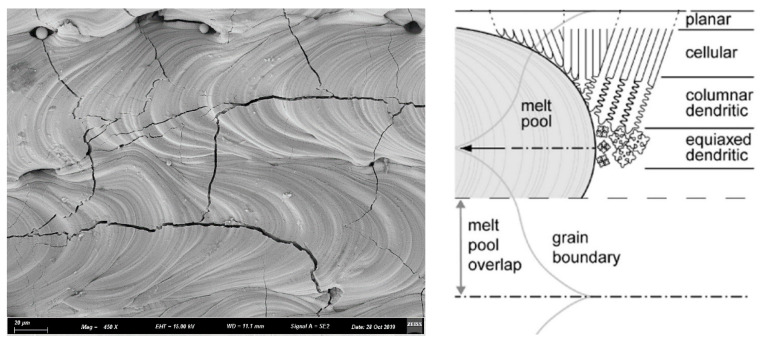
Schematic representation of the microstructural development during SLM.

In the molten pool, the direction of grain growth is opposite to the direction of the temperature gradient. As shown in Figure 12, since the molten pool is elliptical, the direction of temperature gradient has a rotation, which also change the direction of grain growth. This is because there is a large positive temperature gradient at the front edge of the solid-liquid interface, which inhibites nucleation. Therefore, the solidified microstructure forms in an epitaxial growth mode. In SLM Mo parts, randomly orientated flame-like grains growing across the deposition layers were observed, as shown in Figure 7. It suggests that for Mo the rotation of the heat flux had a greater effect than the competitive grain selection during solidification.

**Figure 13:**
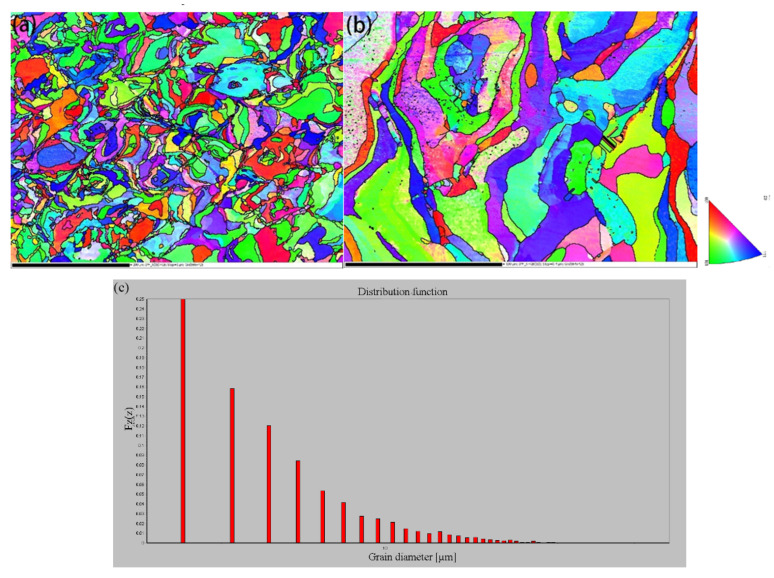
EBSD images of: (**a**) top view, (**b**) side view of SLM fabricated sample, (**c**) grain size distribution data statistics.

EBSD images were used to visualize the grain structure and to determine the local texture of the materials. As shown in Figure 13, a disorder grain morphology with pronounced texture was observed in the top view of the cross section, and equiaxed grains was distributed randomly. In the side view of cross section, larger columnar grains were observed, meaning fast cooling conditions. During the SLM process, the epitaxial growth mechanism usually occur, which means that the grains grow on the base of a previous layer without altering the crystallographic orientation. In contrast, the laser scanning direction was rotated 67° between layers, and grain boundary structure evolved from columnar to interlaced, as shown in Figure 13b. As reported in many articles, columnar grains are formed in SLM. Columnar grains grow in the direction of the building towards the melt pool due to the direction of heat transfer. The columnar grains span several layers and facilitate bonding between layers. Due to the method of bonding between layers, fractures and defects are usually present between layers, which can be reduced by stretched columnar grains. Stretched columnar grains reduce this weakness caused by interlayer bonding. The average grain size calculated from the top view EBSD data is less than 10 μm (Figure 13c), which is much smaller than the traditionally processed method of molybdenum.

The SLM fabricated Mo samples showed superior hardness compared with PM processed Mo (160–180 HV). The reasons are explained below. First, as the laser power increases, grain refinement leads to fine grain strengthening. The nanosized sub-grains (Figure 8d) would be formed by rapid solidification at the extremely high cooling rates that occur in the laser process. The cooling rate in the laser forming process can reach 1010 K/s, and the high thermal conductivity of Mo (142 W/mK) accelerates cooling, which would further refine the grain. The smaller the size of grain, the more grain boundaries are presented. The obstruction of the dislocation motion also leads to an increase in deformation resistivity.

Second, there is large residual stress produced bt the SLM procedure, which has a positive effect on the hardness and strength of the sample, because a reasonable level of residual stress in SLM-processed parts may cause dislocation strengthening and hardness enhancement. Meanwhile, the location of the measuring point also affects the hardness values, as the hardness at the edge of a crack or pore is very low. Therefore, the hardness and strength can be improved effectively by restraining the defects, such as pores and cracks.

Pure molybdenum processed via SLM has columnar grains, equiaxed grains, cracks and pores, and generally shows a complete intergranular fracture surface. Processing molybdenum powder with an atmosphere of low oxygen content of lower than 200 μg/g and controlling the process maps in an appropriate zone, as the temperature is well above the DBTT, does not suppress these defects entirely and does not lead to a changed fracture mode.

## 5. Conclusions

In conclusion, high-density pure molybdenum was prepared by Selective Laser Melting with optimized processing parameters. The main conclusions are summarized as follows:

Spherical powder and optimized processing parameters were necessary for the SLM of pure molybdenum. The shrinkage of the polygon powder was two times that of the spherical powder. A reasonable match of these factors was beneficial in order to achieve good performance. The processing window could be divided into four distinguishing zones depending on the quality of the melting tracks, including weak sintering zone, unstable melting zone, continuous track zone and excessive zone. In the continuous track zone, a relatively smooth track was obtained with an LED interval of 0.44 J/mm to 0.64 J/mm. The highest density of pure molybdenum obtained by SLM forming is 99.1%

A dense microstructure with few microspheres and microcracks was formed in pure molybdenum samples, and cracks could be distinguished into longitudinal and transverse cracks. We found that balling and cracking can be reduced by adding a support structure and suppressing the oxygen content. The hardness of SLM-fabricated molybdenum exceeding 260 HV, which is 30–37% higher than Mo prepared by conventional manufacturing methods, was mainly due to fine grains and dislocation strengthening in the SLM process. The bending strength of SLM-ed Mo reached 280 ± 52 Mpa. Pure molybdenum processed via SLM has columnar grains and equiaxed grains, and generally shows a fully intergranular fracture surface. This study provides a new route for the fabrication of refractory metals of a complex structure.

## Figures and Tables

**Table 1:** The chemical composition of spheroidized Mo powder.

Elements	Mo	Al	Si	Cr	Fe	Cu	O	Others, Total
Results wt(%)	≥99.9	≤0.01	≤0.01	≤0.01	≤0.01	≤0.01	≤0.03	≤0.02

## Data Availability

All data generated or analyzed during this study are included in this published article.
